# In Vivo Study on the Safe Use of a Novel Intraoperative Sensing Tool for Tissue Stiffness Assessment in Endoscopic Surgery

**DOI:** 10.3390/bios15090581

**Published:** 2025-09-05

**Authors:** Georgios Violakis, Pantelis Antonakis, Emmanouil Kritsotakis, Theodoros Kozonis, Leonidas Chardalias, Apostolos Papalois, Georgios Agrogiannis, Effrosyni Kampouroglou, Nikolaos Vardakis, Stylianos Kostakis, Eleni Athanasaki, Zhenyu Zhang, Martin Angelmahr, Manousos Konstadoulakis, Panagiotis Polygerinos

**Affiliations:** 1 Department of Electrical and Computer Engineering, School of Engineering, Hellenic Mediterranean University, 71004 Heraklion, Greece; nickolasvardakis2013@gmail.com; 2 Componous P.C., 11 Kontopoulou Street, 53100 Florina, Greece; kostakiss@componous.gr (S.K.); athanasakih@componous.gr (E.A.); 3 2nd Department of Surgery, Aretaieion University Hospital, National and Kapodistrian University of Athens (NKUA), 11528 Athens, Greece; pantonakis@med.uoa.gr (P.A.); ekritsotakis@aretaieio.uoa.gr (E.K.); lchardal@med.uoa.gr (L.C.); apapalois@med.uoa.gr (A.P.); effkamp@uoa.gr (E.K.); mkonstad@med.uoa.gr (M.K.); 4 Laboratory of Experimental Surgery, Aretaieion University Hospital, National and Kapodistrian University of Athens (NKUA), 11528 Athens, Greece; 5 1st Department of Pathology, Medical School, National and Kapodistrian University of Athens, 11527 Athens, Greece; 6 Department of Fiber Optical Sensor Systems, Fraunhofer Heinrich Hertz Institute, Am Stollen 19H, 38640 Goslar, Germany; zhenyu.zhang@hhi.fraunhofer.de (Z.Z.); martin.angelmahr@hhi.fraunhofer.de (M.A.); 7 Control Systems and Robotics Laboratory, Department of Mechanical Engineering, School of Engineering, Hellenic Mediterranean University, 71004 Heraklion, Greece; polygerinos@hmu.gr

**Keywords:** minimally invasive surgery, stiffness mapping, fiber Bragg grating, palpation

## Abstract

A novel endoscopic palpation tool (EPT), designed for tactile and stiffness sensing using fiber Bragg gratings (FBGs) was evaluated in a surgical environment for intraoperative safety and effectiveness. The EPT consisted of four FBGs arranged in a cross pattern and embedded within an elastic, hollow, silicone hemispherical dome designed to deform upon contact with soft tissue. The EPT was employed to scan both in vivo and ex vivo tissue samples. Monitoring of porcine vital signs during minimally invasive and open surgical procedures showed no significant changes during use of the EPT. Perioperative blood tests including inflammatory markers and liver and renal function studies were unremarkable. Histopathological analyses of tissues involved (liver, spleen, bowel, and abdominal wall) showed no evidence of inflammation, necrosis, or tissue damage, confirming the device’s biocompatibility. To the best of our knowledge, this is the first study reporting in vivo stiffness measurements using an FBG-based EPT. The probe successfully distinguished between soft and hard tissue regions’ relative stiffness. Furthermore, successive measurements on liver samples demonstrated the device’s ability to generate stiffness maps, enabling clear visualization of spatial variation in tissue stiffness.

## 1. Introduction

Minimally invasive surgery (MIS) procedures, both laparoscopic and robotic, offer patients significant advantages compared to open procedures, including expedited functional recovery, less postoperative discomfort, and decreased surgical trauma complication rates. Despite major advancements in technology, these methods still face serious limitations, mainly the absence of haptic feedback, which is an important advantage of open surgery [1,2]. MIS approaches lack the natural real-time assessment of tissue stiffness and adherence to surrounding organs via the surgeon’s hand(s), since all tactile information is indirect and limited to what one can “feel” from the interaction between the tip of the laparoscopic or the robotic instrument and the tissue. This becomes relevant especially in MIS oncological procedures, when one needs to decide if and which tissue needs to be sent for frozen section biopsy during initial laparoscopy or when trying to determine the extent of resection margins.

To address this limitation, the development of tactile sensors for MIS applications has recently garnered significant research interest [3,4]. Various tactile sensing technologies have been explored, including imaging-based sensors [5,6], some of them utilizing imaging probes with IR-cut filters for increased accuracy that can be easily retrofitted in existing endoscopic probes [7]. Another technology utilized for tactile sensing is based on electrical resistance-based sensors [8], and force sensitivity down to 0.05 N has been achieved using capacitive sensors [9,10]. Piezoelectric sensors have also gained significant traction, as they can be packaged in very small sizes suitable for MIS [11]. A review of the main electrical signal based tactile sensors can be found in the work of Chi et al. [12], which also includes several optical-based tactile sensors. Recently, novel magnetic materials based tactile sensors have been proposed, however, their current implementation requires a relatively large surface area that would be unsuitable for MIS [13]. In another work by Yue et al., endoscopic optical coherence tomography is utilized for micrometric geometric deformation and contact force measurement, albeit without any in vivo or phantom characterization [14]. Optical tracking methods have also been proposed in various implementation designs, providing very good resolution [15,16].

Among the various sensors, photonics-based sensors that utilize fiber Bragg gratings (FBGs) have gained particular attention. These sensors are capable of precisely monitoring a wide range of physical parameters such as temperature, strain, pressure, or deformation [17,18,19,20]. Their high sensitivity, compact form, and immunity to electromagnetic interference make them especially attractive for MIS.

Several FBG-based tactile sensors have been developed by integrating FBGs with micromachined mechanical structures [21,22]. Other FBG-based sensors have been demonstrated that are either flexible [23] or mounted on forceps [24], and non-FBG based tactile sensors have also relied on acoustic reflections [25], but none of these have been tested in vivo. A recent design proposed the use of two series connected FBGs for tactile sensing, separated using a spring, housed inside an endoscopic enclosure [26], which was evaluated only ex vivo. This sensor achieved a resolution of ~0.02 N at a 1 N dynamic range. Similarly, a dual FBG-based Fabry-Pérot (FP) cavity-based sensor designed for MIS was used for in vitro experiments simulating tumors [27], achieving a sensitivity of 124.75 pm/N (or ~0.096 N resolution for a typical 15 pm FBG interrogator resolution that can increase to 0.016 N with a 2 pm high resolution interrogator).

Despite their promising performance, overall compactness, robustness, and resilience, their use in real-life surgical settings requiring repetitive insertions through surgical trocars has not yet been conclusively demonstrated. Recently, a simplified FBG-based tactile sensor design was described, featuring arrays of FBGs inscribed in polymer optical fibers [28]. This design utilized neural network algorithms for data analysis and achieved a sensing resolution of 0.07 N.

Based on the positive results of a previously developed tactile sensor using FBG sensors embedded within a silicone dome at the tool tip [15], the aim of this study was to validate the safety, the performance, and the resilience of a novel endoscopic palpation tool (EPT) with this sensor, in actual surgical procedures in an animal model.

## 2. Materials and Methods

### 2.1. Palpation Probe Design, Manufacturing, Monitoring and Materials

**Figure 1:**
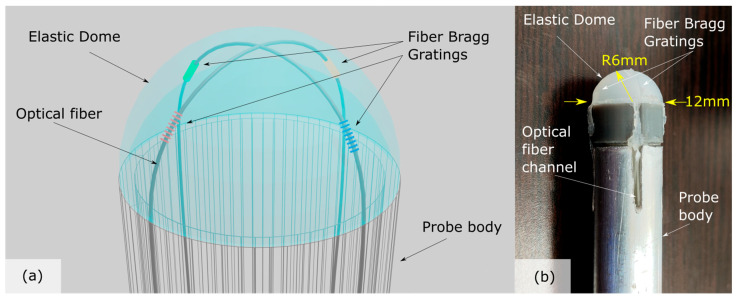
(**a**) Palpation probe schematic layout comprising an elastic membrane of hemispherical shape and a single optical fiber with 4 fiber Bragg gratings in cross-linked configuration; (**b**) Picture of the produced probe head.

The endoscopic palpation tool (EPT)’s principle of operation is based on the online deformation monitoring of an elastic membrane of a hemispherical shape (dome). Dome deformation data are collected via an embedded single optical fiber cable containing fiber Bragg gratings that act as point mechanical strain gauges. As the dome encounters the tissue, it deforms under the forces exerted on it and this deformation is then manifested as Bragg wavelength shifts. These spectral displacements are then used to extract relative stiffness values of the target tissue. A conceptual sketch of the probe head is presented in Figure 1a, along with a picture of the fabricated probe head in Figure 1b.

**Figure 2:**
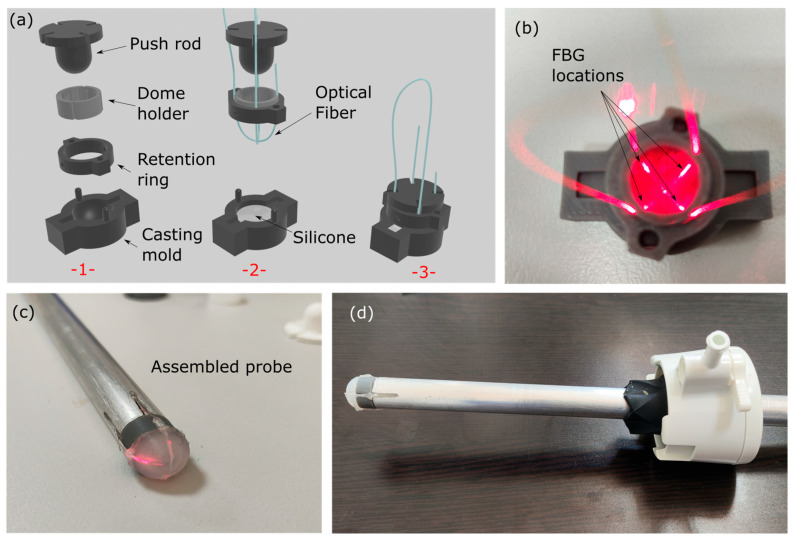
(**a**) Hollow dome with optical fiber fabrication process; (**b**) Optical fiber with 4 FBGs (see scattered red light) inside the casting mold; (**c**) Assembled probe head with FBG positioning check (red lights) before sealing; (**d**) Assembled palpation probe through a 15 mm trocar port.

The total diameter of the probe is 12 mm as it is intended to be passed through 12 mm or 15 mm ports. The hemispherical dome has a total thickness of ~1 mm and is fabricated using the mold casting technique (Figure 2a). Briefly, a 12 mm probe ‘negative’ of the dome was manufactured using fused deposition molding 3d printing which included a separate part for placing the optical fiber and a pressing element to form the concave shape (Figure 2a(-1-)). A 2-component liquid elastic silicone (EcoflexTM 00-20 hardness silicone rubber, Smooth-On, Macungie, PA, USA), which solidifies after mixing, was used for the membrane and placed inside the casting mold (Figure 2a(-2-)). The optical fiber was positioned in such a way that it intersected within the top of the dome forming an ‘X’ shape (Figure 1a and Figure 2a(-2-)). The casting process was terminated by pushing the liquid silicone through a hemispherically tipped push-rod in order to form the hollow dome (Figure 2a(-3-)). The mold, the retention ring, and the push rod were removed after silicone curing. The optical fiber terminated inside the supporting body, which was an aluminum tube with an outer diameter of 12 mm and an inner diameter of 9 mm (Figure 2b–d). The optical fiber was a standard telecommunications SMF-28e G.652.D compliant optical fiber (8.2 μm core diameter, 125 μm cladding diameter and 250 μm acrylic coating diameter). As evidenced in Figure 2a,b, the position of the FBG sensors could be determined both before and after fabrication by monitoring the red scattered light emitted by the area where FBGs were inscribed.

Femtosecond laser pulses were utilized to induce uniform, periodic modulations in the refractive index of the optical fiber, thereby forming FBG structures. The grating inscription was performed using a commercially available femtosecond laser system (Ti:sapphire Tsunami/Spitfire Pro, Newport Spectra-Physics GmbH, Darmstadt, Germany), which emitted laser pulses with a central wavelength of 800 nm and a pulse duration of 90 fs [29]. For precise spatial control during the FBG inscription process, the optical fiber was securely mounted on three high-precision translation stages (Physik Instrumente, Karlsruhe, Germany), allowing accurate positioning in three dimensions. The laser pulses were focused into the core of the fiber using a 40× objective lens (Carl Zeiss AG, Oberkochen, Germany), ensuring a high-intensity focal spot necessary for nonlinear absorption and refractive index modification in the fiber core. The pulse energy was set to 81 nJ at a repetition rate of 10 Hz, measured below the objective lens. Multiplexing of the FBGs was achieved by inscribing multiple gratings at distinct positions along a single optical fiber. To ensure uniform spatial distribution of FBG sensing points on the three-dimensional tip of the silicone dome, the FBG array was organized into two pairs. Within each pair, the FBGs were separated by 15 mm, while the distance between the two pairs was set to 100 mm.

In total, 4 sensors were inscribed, positioned near the base of the elastic dome in order to provide vectorial pressure information on the dome’s deformation, by monitoring the relative Bragg peak shifts. The cast dome and the aluminum probe body were assembled together using specially designed joints, one for accommodating the dome itself and another for mechanically coupling the dome and the aluminum probe body. At the tip of the probe body, four openings were machined to facilitate optical fiber cable management. At the end of the assembly process, these openings were sealed using silicone glue.

**Figure 3:**
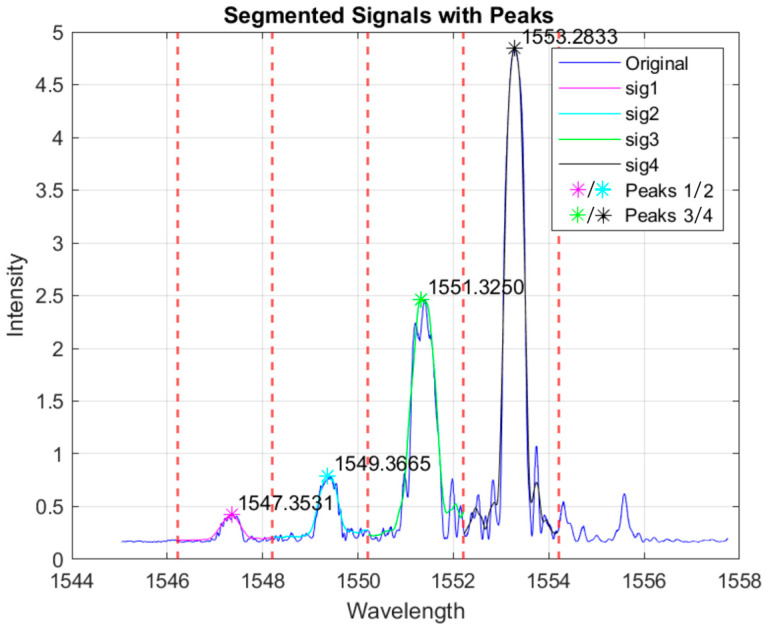
Spectral output of the probe without any pressure applied on the probe head. Signals are fitted for precise determination of the central Bragg peak wavelength.

The FBG sensors were monitored using a single-channel fiber Bragg grating interrogator (Componous P.C., Florina, Greece) providing a hardware spectral resolution of 2 pm (increased through peak fitting algorithm to below 0.1 pm) and a wavelength stability better than 1 pm over 5 h of continuous operation. A non-normalized spectral output of the monitoring system in resting position (without pressure on the membrane) is presented in Figure 3. The spectral data were interrogated and plotted at a rate of 10 Hz providing effectively real-time output of the raw signal data. Although the FBGs were inscribed with a similar reflectivity of ~30%, the non-linear optical power output of the interrogator produced the discrepancy between the peak amplitudes. The FBG interrogator was not operated at the maximum optical power output; therefore, the aforementioned effect can be compensated by updating the device software with a power compensation algorithm that outputs more optical power towards the blue side of the spectrum. As a result, the peak fitting process will be equally precise for all four peaks without biasing favorably the response of the peaks at the longer wavelengths. For the analysis in this work, only the Bragg wavelength shifts were used to estimate stiffness, without considering changes in the amplitude of the peaks.

Using software, each Bragg grating spectrum is divided into four individual spectral parts (red dashed lines) where each of the four Bragg gratings is expected to move. Then, a Gaussian function fitting procedure takes place in order to acquire the Bragg peak of each grating, which is derived from the parameter defining the Gaussian function center. This procedure allows the determination of the Bragg peak position at an accuracy higher than that allowed by the hardware itself (2 pm).

Temperature compensation did not take place during the experiments, despite the known sensitivity of FBGs to temperature. This decision was taken based on the controlled environment of the surgical room. No temperature changes were expected in the ex vivo scans and similarly, steady conditions were expected inside the porcine body for the in vivo measurements. Future versions of the probe head will nevertheless include a temperature compensation FBG at the end of the terminated optical fiber inside the probe head but detached from the dome itself.

**Figure 4:**
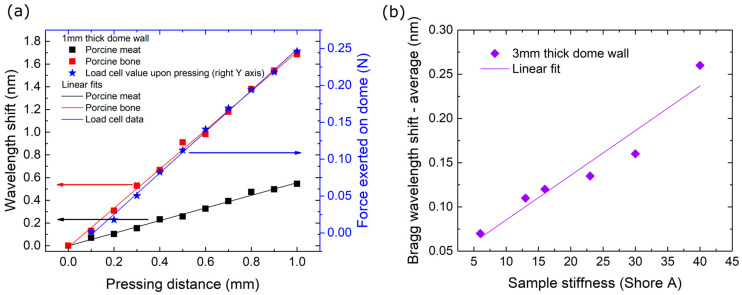
(**a**) Average Bragg wavelength shift response versus a soft and a hard target. Second y-axis: Force exerted on elastic dome of 1 mm thickness for various pressing distances. Colored arrows correlate data points to the corresponding y-axis (red and black squares: left y-axis, blue stars: right y-axis) (**b**) Probe head sensitivity versus targets of varying Shore A hardness (3 mm thick wall dome).

The symmetrical placement of the four FBGs around the dome with a 90-degree angle between them allows the determination of both the exerted force amplitude and direction. However, such calibration would require numerical calculations and remains outside the scope of the presented work. Nevertheless, a series of measurements was carried out in order to estimate the sensitivity and linearity of the probe head and also to estimate the effect of design parameters such as wall thickness. Initially, the probe used in this work was pressed against a load cell with a dynamic range of ~1 N (100 g). The probe was placed normally to the load cell and pressed against at predefined positions to determine wavelength shift response versus indentation distance. The results are presented in Figure 4a.

The hemispherical wall thickness, diameter, and material, as well as the optical fiber material, diameter, and bend radius are all factors affecting the sensitivity and dynamic range of the probe head. The design choices that were taken in this work were realized under the assumption that forces less than 1 N would be exerted on the probe as it palpates over soft tissue. In order to acquire a first, draft, overview of the effect of one design parameter, specifically, the wall thickness, a series of silicone targets of predefined shore hardness was prepared and a 3 mm wall thick probe was pressed against these at an indentation distance of 2 mm. The results are presented in Figure 4b. The probe head exhibited a linear response within this range, although the Bragg wavelength shifts were evidently smaller than the shifts observed with the thinner wall probe (Figure 4a). A full calibration of the probe including vectorial information will follow in a future work.

### 2.2. Anesthesia and Surgical Technique

All procedures and measurements were performed at the Experimental Surgery Laboratory of Aretaion Hospital (Laboratory License: EL 25 BIO exp 024, Protocol License: No. Pr. 1044199/20-10-2022).

The study was performed in 2 healthy landrace/large-white pigs (male sex, 16 weeks old, average weight, 30 +/− 2 kg) under general anesthesia. The sole selection criterion was that the animals were healthy. The animals were acclimatized in our lab for 1 week before experimentation in adjacent cages. Sample size was decided considering the availability of animals and the purpose of the study, namely, the safety and efficacy of the device. No sample size calculation was performed.

All animals were pre-medicated using midazolam (0.57 mg/kg body weight [B.W.]) and ketamine (15 mg/kg B.W.), which were both administered intramuscularly (i.m.), and atropine (0.045 mg/kg B.W.) was administered endotracheally 10 min before intubation. The intubation was performed with propofol (3 mg/kg), fentanyl (0.012 mg/kg), and cisatracurium besylate (0.5 mg/kg), which were administered as an intravenous (IV) bolus via an ear vein.

General anesthesia was provided using propofol (9 mg/kg/h), and sedation was achieved using fentanyl (2 mg ¼ 4 amp in 500 mL of 0.9% saline) and cisatracurium besylate (200 mg ¼ 10 amp Nimbex in 500 mL of 0.9% saline) at 60 to 80 mL/h IV, which was continuously administered via an ear vein. The animals remained intubated and mechanically ventilated using 40% FiO_2_ at 20 breaths/minute (Drager anesthesia machine).

All animals’ vital signs were closely monitored during the procedure (arterial pressure, oxygen saturation, and cardiac rate).

Following entrance to the peritoneal cavity with Hasson’s technique at the periumbilical area, pneumoperitoneum was created and maintained at 12 mm Hg. Next, a 15 mm trocar was inserted under direct vision in the right lower quadrant, which was used for EPT insertion, and another 5 mm working port in the left lower quadrant to facilitate tissue exposure and manipulation. The novel EPT was applied sequentially to the surface of the liver, spleen, small intestine, and the left costal margin. At each point, measurements were first made just before and during light contact with the tissue. The area of EPT contact with the tissue was marked with an acrylic clip placed immediately right to it to ensure accurate tissue sampling later. Blood samples were drawn from a central venous line before and after EPS application for analysis. Tissue areas where the EPT was applied along with the adjacent ones (acting as control samples) were sent for pathology review to establish necrosis and hyalinosis.

### 2.3. EPT Physiological Effects—Safety Assessment

- Cardiopulmonary function: To assess safety and the potential effect of intraoperative EPT application to the cardiopulmonary function, basic physiologic parameters including blood pressure, pulse rate and SpO_2_ were recorded before the initial and after the final application of the EPT.
- Inflammatory response, liver and renal function: Blood samples were drawn before the initial and after the final application of the EPT during laparoscopy to assess safety and its potential effects on inflammatory response and liver and renal function. White blood cell count, neutrophils, and C-reactive protein were used for the assessment of the inflammatory response, aspartic (AST) and alanine (ALT) aminotransferase for liver function, and urea and creatinine levels for renal function evaluation.
- Histopathological assessment: After the completion of laparoscopy, a midline laparotomy was performed. All areas of EPT—tissue contact (left side of the acrylic clip) were retrieved along with the neighboring tissue on the right side of the clip, the latter serving as normal control tissue. After initial formalin fixation all samples were evaluated in hematoxylin—eosin-stained sections for the following parameters: inflammation, necrosis, and stromal changes (hyalinization and changes in vessel wall).

### 2.4. EPT Performance in the Assessement of Solid Objects Underneath Normal Liver Parenchyma

During laparotomy, a 4 cm × 4 cm area was marked on the left liver lobe of each animal. Through a small incision, a metallic blade (surgical scalpel or stitching needle) was inserted underneath this area and EPT measurements were recorded on the marked liver surface above and around the blade. This was carried out to assess the EPT’s ability to differentiate between liver parenchyma with and without a solid structure under it, trying to imitate the existence of a deep metastatic lesion under normal-appearing liver parenchyma.

## 3. Results

### 3.1. EPT Effects and Safety Profile

The EPT’s effects on the vital signs of the swine as well as the tissue it came in contact with are summarized in Table 1.

#### 3.1.1. Cardiopulmonary Function

The application of the EPT did not compromise the cardiopulmonary function of either animal model. In both subjects, the heart rate showed a minor increase following the procedure, from 110 to 130 beats per minute (bpm) in Swine 1 and from 120 to 129 in Swine 2. Systolic and diastolic pressure were also not affected, both swine remained stable with a pressure of 117/90 mmHg before the procedure to 110/93 after in Swine 1 and 125/95 mmHg to 110/85 mmHg in Swine 2. Peripheral oxygen saturation (SpO_2_) remained stable during the procedure, ranging from 95% to 97% in both animals. Body temperature also remained unaffected across all time points. These findings suggest that the use of the EPT did not adversely affect the pulmonary or hemodynamic parameters of the subjects.

#### 3.1.2. Inflammatory Response, Liver and Renal Function

In terms of inflammatory response, a mild increase in WBC was observed in both swine, from 5900 to 9600/mm^3^ in Swine 1 and from 6200 to 6400/mm^3^ in Swine 2. Neutrophil percentage also moderately rose from 36% to 52% in Swine 1, and 41% to 47% in Swine 2. C-reactive protein (CRP) remained unchanged at 0.06 mg/dL in both models. These results demonstrate that no acute inflammatory reaction was triggered by the EPT’s application.

Hemoglobin values and platelet count remained constant pre and post intervention, with no apparent intraoperative blood loss. This was also the case for liver function tests. Liver enzyme levels demonstrated slight postoperative elevations in Swine 1, with AST increasing from 46 to 64 IU/L and ALT from 58 to 61 IU/L. In Swine 2, both AST and ALT values decreased slightly, from 159 to 148 IU/L and from 67 to 60 IU/L, respectively.

Renal function measurements showed minimal alterations with urea levels remaining within acceptable limits 32.2 to 34.1 mg/dL in Swine 1 and 34.2 to 31 mg/dL in Swine 2. Creatine values remained stable at 1.4 mg/dL in Swine 1 with a mild increase observed in Swine 2 from 2.2 to 2.4 mg/dL.

#### 3.1.3. Effects on Tissues Histology

Histological evaluation was performed on tissue specimens from the liver, spleen, bowel, and abdominal wall, obtained from areas directly subjected to EPT contact and pressure as well as from adjacent control sites. Comparative histological evaluation did not reveal any alterations with preserved parenchymal and stromal architecture and with no evidence of acute inflammation, necrosis, stromal fibrosis, or hyalinization.

### 3.2. Stiffness Mapping Results

In this section, the performance of the sensor in determining stiffness changes is described. The device itself works by pushing the elastic dome towards the soft tissue of interest. For the evaluation protocol, the tip of the device (the tip of the dome) was brought into contact with the soft tissue and a full spectrograph of the four peaks was recorded. Then, the surgeon pushed gently towards the soft tissue and a second spectrograph was acquired. The relative Bragg peak positions of all four FBG peaks were then extracted and compared to create a relative peak displacement before and after measurement.

#### 3.2.1. Endoscopic Palpation Scans

**Figure 5:**
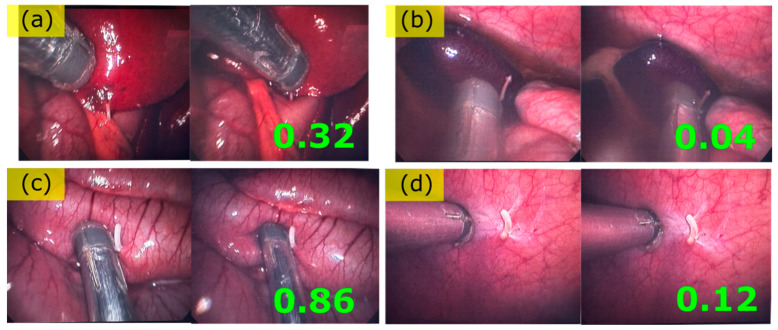
Endoscopic palpation tool scans of (**a**) liver, (**b**) spleen, (**c**) bowel, (**d**) abdominal wall. The green numbers demonstrate a relative stiffness value extracted by pressing the probe towards the tissue under examination.

The series of porcine organs that were endoscopically palpated using the EPT is presented in Figure 5. For every tissue examined from Figure 5a–d, the procedure followed for the stiffness measurement was the same. A first contact to the tissue was established while ensuring no or minimal Bragg wavelength shift incurred upon this first contact (left part of images pair throughout Figure 5a–d). A Bragg peaks spectrum was saved at this point. Then, after a ~2 mm deep motion towards the tissue, a second spectrum of the Bragg peaks was acquired (right part of images pair throughout Figure 5a–d).

In the next step, the acquired spectra were analyzed in order to acquire the Bragg peak position before and after the palpation scan motion. Once the Bragg peak position was acquired, the relative Bragg peak shift *Δλ_B_* was determined for each channel, namely, the Bragg peak shift before and after pushing the sensor towards the soft tissue. Then, the absolute values of each channel’s Bragg peak shifts $\left|{\Delta \lambda }_{B}\right|$ were summed together for all four Bragg peaks in order to establish a first non-vectorial approximation of the tissue’s relative stiffness upon pressure under constant displacement. The results were normalized to a value of 1 for the stiffest tissue and 0 for the softest. The results are displayed as a green number on the lower right-hand side of the image pairs in Figure 5. However, it is worth noting that the vectorial information provided by monitoring the four Bragg peaks was not exploited in this first approach. Additionally, it should also be noted that there was no strict control over the overall displacement of the probe towards the soft tissue.

#### 3.2.2. Liver, Ex-Vivo, Scan 1

**Figure 6:**
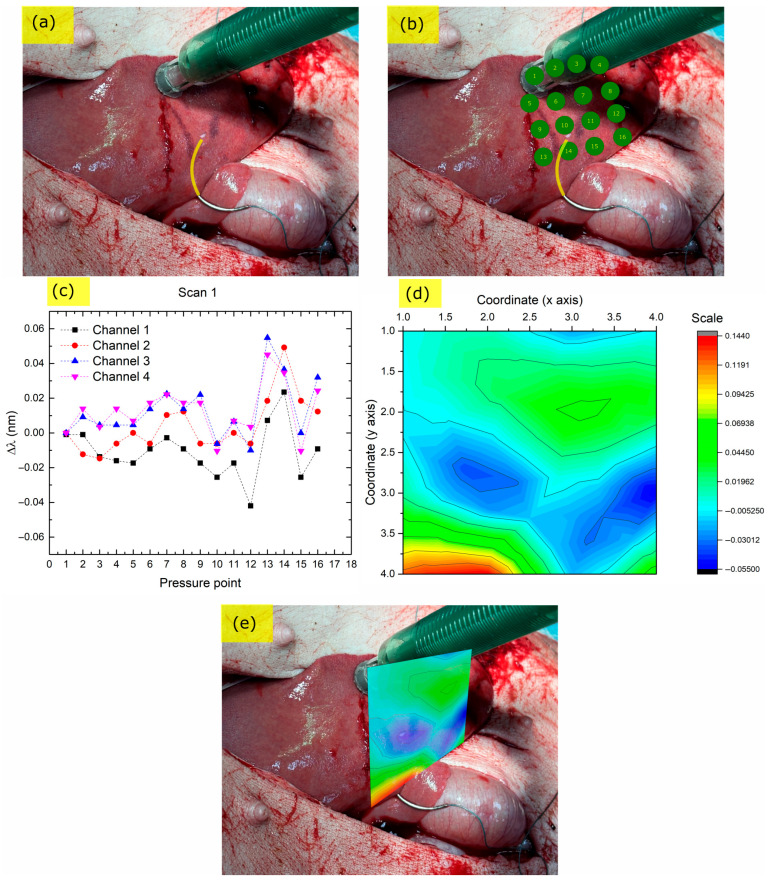
EPT scan of liver using a stitching needle as a control point. (**a**) Liver with underlying stitching needle (yellow marker); (**b**) Green points: EPT pressing points in sequential order; (**c**) Bragg peak shift response at each pressing point; (**d**) Interpolated relative stiffness map (red color: higher stiffness, blue color: lower stiffness); (**e**) Stiffness ‘heat map’ overlay on liver.

The palpation measurements on the liver surface with the addition of a hard control object in the underlying parenchyma (stitching needle) are presented in Figure 6.

The yellow highlighted area in Figure 6a shows the penetration depth of the needle below the liver tissue. Figure 6b shows the points where palpation measurements were taken by pressing the probe towards the liver in sequential order. The EPT tip position was determined from sequential images provided by the surgeons, using a custom algorithm that locates the tip of the probe on the image and adds a marker at the contact point. In a final implementation of the device, the tip position is expected to be automatically extracted by the combinational use of an endoscopic camera and a built-in accelerometer.

The Bragg wavelength shifts with respect to the first contact point (which was not pressed against the tissue) are presented in Figure 6c for all four Bragg peaks of the sensor head. Τhe elevated response of the sensor when pressed at points 13 and 14, which are next to the stitching needle, is prominent. Similar to the endoscopic scans, the absolute values of each channel’s Bragg peak shifts $\left|{\Delta \lambda }_{B}\right|$ were summed together for all four Bragg peaks and a relative stiffness value was extracted. This value was then overlaid on a matrix forming the pressure points of each measurement, creating a contour map with the *z* axis being the relative stiffness value and the *x* and *y* axes the relative x, y coordinates, as shown in Figure 6d. The contour map shows the difference between parenchyma with the needle (red color) and without it (green color).

A projected image of the stiffness ‘heat’ map right above the tissue area is presented in Figure 6e. Of note is that this off-line image projection can be easily implemented in the interrogator software for online monitoring and heatmap creation during the surgery given that the probe tip coordinates are acquired in an automated fashion.

In general, it was observed that the EPT could easily discriminate between soft liver tissue and the stitching-needle-affected area, as shown by the Bragg wavelength shifts (Figure 6c) and the corresponding contour plot (Figure 6d,e). Outside the stitching needle area, around points 5 to 9, an elevated stiffness was observed which could, however, be the result of harder or softer pressing towards the tissue, highlighting the need for precise pressing distance towards the tissue under test.

#### 3.2.3. Liver, Ex Vivo, Scan 2

**Figure 7:**
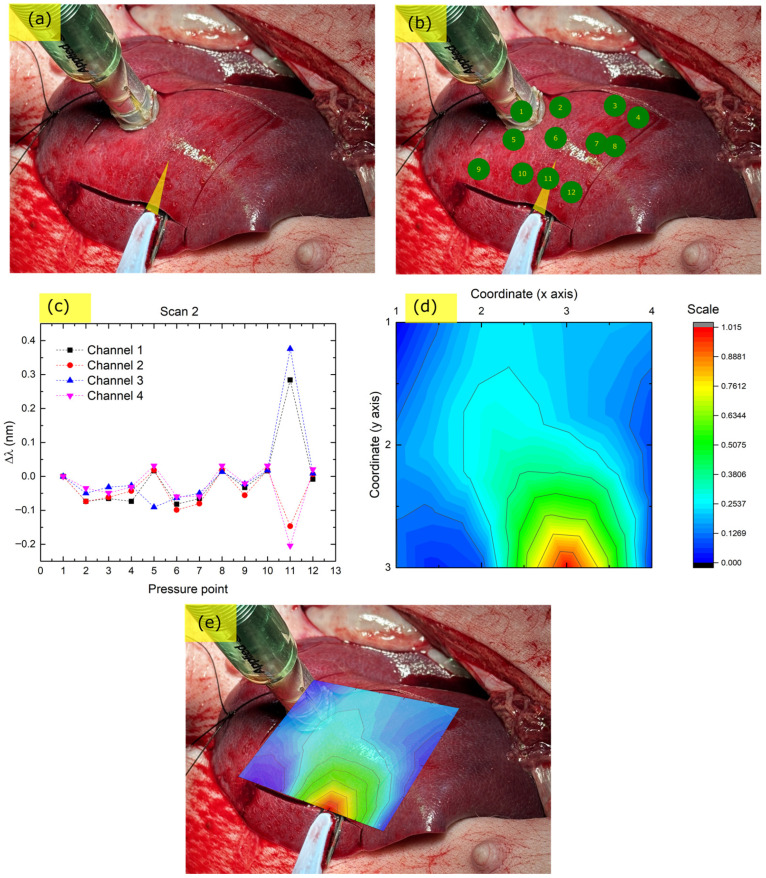
EPT scan of liver using a scalpel as a control point and under direct pressure of the probe over the point with the hard target. (**a**) Liver with underlying scalpel (yellow marker); (**b**) Green points: EPT pressing points in sequential order; (**c**) Bragg peak shift response at each pressing point; (**d**) Interpolated relative stiffness map (red color: higher stiffness, blue color: lower stiffness); (**e**) stiffness ‘heat map’ overlay on liver.

In order to further assess the performance of the EPT, a second palpation scan was conducted on the exposed liver, this time with a scalpel underneath the soft tissue (Figure 7a). Additionally, care was taken to come directly above the submerged scalpel with the EPT probe head when performing the measurement, in order to examine the differences between direct and side contact with hard targets.

The series of palpation measurements and the pressure liver areas are presented in Figure 7b. The corresponding Bragg wavelength shift responses are presented in Figure 7c and the sum of the absolute values of these shifts was used to create the stiffness ‘heat’ map contour plot in Figure 7d. This stiffness map is overlaid on the liver in Figure 7e.

Unlike the stitching needle example in Section 3.2.2, here, the direct contact of the probe head provoked a much larger change in amplitude of the Bragg peaks’ position with a similar pressing depth of the tool. Specifically, is the response was almost an order of magnitude stronger than that of the stitching needle in the previous section. This difference can be attributed to the direct contact of the probe head with the hard target, whereas in the case of the needle, the contact was indirect from the side of the probe. This change in response amplitude highlights further the need to proceed with vectorial analysis of the acquired signals in order to achieve more accurate stiffness maps. In addition, vectorial analysis can also pinpoint hard target position with respect to the dome, which increases spatial resolution.

Of note is that depending on the probe angle of incidence towards the tissue, the Bragg peaks can undergo either blue- or red-shifts, due to compressive or tensile stresses. At the same time, under larger deformations the peaks undergo amplitude changes, due to bend losses and scattering losses due to the FBG fabrication method. This combined wavelength and amplitude information can be used to provide rasterized data on the stiffness of the tissue at a resolution that is much higher than the one acquired by the simplified method in the current work. For example, under a deformation that caused a wavelength shift of 1.6 nm to two of the Bragg peaks of the sensor, an amplitude reduction of ~28% was observed, which was more sensitive to changes than the spectral shifts. Preliminary sensitivity measurements that take amplitude into account exhibit a three-fold sensitivity increase compared to just monitoring the spectral shifts, because of the higher sensitivity to bending/scattering losses and the higher = resolution 16-bit analog-to-digital signal conversion of the amplitude signal. However, due to the complex calibration required for such a full-vectorial data acquisition, this functionality will be tested in future work.

## 4. Discussion

The present preclinical study was conducted to assess the intraoperative safety and functional effectiveness of a novel endoscopic palpation tool (EPT) incorporating fiber Bragg grating (FBG) sensor technology embedded inside an elastic hemispherical silicone membrane while also exploring its physiological impact and its ability to yield tactile feedback in a real-time frame during minimal invasive procedures. To our knowledge, this is the first in vivo study using this type of device in live porcine models.

In terms of safety, the results were satisfactory. The effects on cardiopulmonary function, inflammatory response, liver and renal failure were minimal. The histologic findings in the tissues areas where the probe was applied were unremarkable and completely identical to the ones from the neighboring untouched tissue. Therefore, the novel EPT was found to be safe for use in vivo in the laboratory setting, specifically in a porcine model. The next step is to test its safety in real-life surgery circumstances on human tissues.

The second objective of this study was the performance assessment of the device as a palpation or stiffness mapping tool. From a technical standpoint, the EPT delivered consistent stiffness measurements across multiple tissue types and managed to successfully discriminate between different types of soft tissue and identify the location of hard objects underneath the liver parenchyma. The internal FBG-based sensing system was able to detect real-time pressure deformation via shifts in reflected wavelength, which were reliably captured and recorded. The use of such a tool can be envisioned in two different use-case scenarios. The first one is the use of the tool as a hand-held device, as in this study, and the second scenario is the use of the EPT mounted on a robotic surgery platform.

As already demonstrated in this work, it is possible to successfully use the EPT as a hand-held palpation probe. However, it should be noted that as a hand-held device, real-time stiffness mapping of the organ under examination would require either the use of a camera and image analysis (as in this work), or more complex systems that might as well involve accelerometers, gyroscopes, or other positioning hardware on the probe for precise localization. The hand-held probe is also required to be pressed under a consistent distance towards the tissue under testing in order to provide reliable results. This is currently addressed by providing a predetermined pressing depth of 2 mm using a mechanical limiter, but surgeon manipulation can introduce offsets. Under this scope, it is evident that such a device when mounted on a robotic surgery platform would directly benefit from a constant positioning feedback and precise pressing displacement. Future work in this aspect will be directed towards the consistent pressing of the probe towards the soft tissue, regardless of the force applied by the surgeon, and incorporating a miniature gyroscope to map relative motion changes.

Previous attempts at developing novel haptic laparoscopic tools are reported in the review work of Huang et al. [30]. Several technologies have been employed, ranging from piezoelectric sensors [31], mechanical sensors such as the Force Reflecting Operation Instrument (FROI) [32], to pressure sensitive capacitors [33] or triboelectric sensor matrices [34]. Fiber Bragg grating-based solutions have also been presented but these were either tested in vitro [35] or do not provide the required sensitivity due to the installation of the FBG to a mechanically clamped grasper [36]. In contrast, our EPT embeds four FBGs inside an elastic hemispherical silicone dome, providing a miniature, accurate, and robust solution for palpation sensing in MIS combined with a low-cost high-resolution readout FBG unit capable of providing real-time stiffness output. Advances in tunable vertical-cavity surface-emitting laser (VCSEL) technology have allowed the commercialization of high-resolution FBG interrogators such as the one used in this work at a cost below EUR 1,500, a price that makes the technology comparable to or cheaper than other competing solutions in terms of cost. The current 12 mm probe diameter implementation is limited by the stiffness and the bending losses of the 125 μm diameter glass optical fiber. An optical fiber of this radius can be bent to smaller bending radii but the glass stiffness will become dominant in the elastic hemispherical dome deformation and also introduce significant bend losses [37]. However, 125 μm optical fibers can be substituted with commercially available 50 μm diameter fibers, which can lead to further miniaturization of the probe diameter down to 5 mm without significant sacrifices to the elasticity or the losses. In this case, however, an even softer silicone would be required for the dome material to compensate for the smaller volume. Future work will be directed towards the incorporation of real-time full stiffness heat-map plotting from the FBG read-out unit and later, the addition of biochemical sensing at the tip of the dome for combined palpation and chemical testing.

## 5. Conclusions

Our novel endoscopic palpation tool (EPT) incorporating fiber Bragg grating (FBG) sensor technology embedded inside an elastic hemispherical silicone membrane was safe to operate and capable of successfully discriminating between soft and hard structures in vivo in a porcine model in a laboratory setting. Further study of this EPT’s performance in human tissues is warranted.

Furthermore, several modifications including automatic tip positioning, precise pressing distance acquisition, vectorial dome deformation analysis, and additional Bragg peak amplitude change monitoring could improve the EPT’s efficacy in the future.

## Figures and Tables

**Table 1:** EPT safety results summary.

	Swine 1		Swine 2	
Vitals	Before	After	Before	After
HR (bpm)	110	130	120	129
Systolic (mmHg)	117	110	125	110
Diastolic (mmHg)	90	93	95	85
SpO_2_ (%)	96	95	97	95
T (°C)	110	130	120	129
**Laboratory**				
WBC (×10^3^/μL)	5900	9600	6200	6400
Neut (%)	36%	52%	41%	47%
Hb (g/dL)	10.9	11.2	12.6	12.5
PLT (×10^3^/μL)	204	207	175	196
Ur (mg/dL)	32.2	34.1	34.2	31
Cr (mg/dL)	1.4	1.4	2.2	2.4
AST (IU/L)	46	64	159	148
ALT (IU/L)	58	61	67	60
CRP (mg/dL)	0.06	0.06	0.06	0.06

## Data Availability

The raw data supporting the conclusions of this article will be made available by the authors on request.

## Abbreviations

The following abbreviations are used in this manuscript:

EPT	Endoscopic palpation tool
MIS	Minimally invasive surgery
FBG	Fiber Bragg grating
FP	Fabry-Pérot
VCSEL	Vertical-cavity surface-emitting laser
